# Dietary Patterns and Feeding Challenges in Children with Neurodevelopmental Disorders

**DOI:** 10.3390/nu18111668

**Published:** 2026-05-23

**Authors:** Laura Torres-Collado, Manuela García-de-la-Hera, Verónica Company-Devesa, Laura María Compañ-Gabucio

**Affiliations:** 1 Unidad de Epidemiología de la Nutrición (EPINUT), Departamento de Salud Pública, Historia de la Ciencia y Ginecología, Universidad Miguel Hernández (UMH), 03550 Alicante, Spain; l.torres@umh.es (L.T.-C.); manoli@umh.es (M.G.-d.-l.-H.); lcompan@umh.es (L.M.C.-G.); 2 Instituto de Investigación Sanitaria y Biomédica de Alicante (ISABIAL), 03010 Alicante, Spain; 3 CIBER Epidemiología y Salud Pública (CIBERESP), Instituto de Salud Carlos III, 28034 Madrid, Spain; 4 Being + Doing & Becoming Occupational Research Group (B + D + b), Departamento de Patología y Cirugía, Universidad Miguel Hernández (UMH), 03550 Alicante, Spain

Neurodevelopmental disorders (NDDs), including autism spectrum disorder (ASD), attention-deficit/hyperactivity disorder (ADHD), and intellectual disability, comprise a heterogeneous group of conditions that emerge during the developmental period and may affect cognition, communication, motor function, adaptive behavior, and daily functioning [1,2]. These conditions frequently show phenotypic overlap and comorbidity, which contributes to complex clinical presentations and highlights the need for individualized approaches [1,2]. Among children with NDDs, feeding difficulties and restrictive dietary patterns are common and deserve particular attention [3,4,5]. Food selectivity, food neophobia, restricted dietary variety, gastrointestinal symptoms, sensory processing differences, and nutritional inadequacies may coexist and interact, increasing the complexity of assessment and intervention in clinical and community settings [6,7].

The first edition of this Special Issue, “Dietary Patterns: Do Children with Neurodevelopmental Disorders Eat Differently?”, highlighted the importance of understanding eating behavior, dietary assessment, and nutritional interventions in children with NDDs [8]. The contributions published in that edition emphasized food selectivity, anxiety, dietary assessment tools, and the need for individualized and multidisciplinary approaches. Building on that work, this second edition expands the field by integrating new evidence on sensory processing, parental feeding practices, gastrointestinal symptoms, microbiome-related mechanisms, oromotor function, and interventions aimed at improving fruit and vegetable consumption.

The original research articles included in this Special Issue provide complementary perspectives on feeding difficulties across different pediatric populations. In children with ASD, Mirizzi et al. (2025) (Contribution 1) examined food selectivity in relation to sensory processing, gastrointestinal symptoms, parental feeding practices, and mealtime behaviors. Their findings showed greater food refusal, lower dietary variety, more sensory processing difficulties, and more autism-related mealtime behaviors in children with ASD than in typically developing peers. Particularly relevant was the identification of a high-risk subgroup characterized by food selectivity, sensory sensitivities, gastrointestinal symptoms, and more controlling parental feeding practices. This multidimensional approach reinforces the idea that selective eating should be interpreted as part of a broader clinical phenotype shaped by sensory, gastrointestinal, behavioral, and family-level factors.

This relationship between sensory processing and diet is also addressed by Olson et al. (2025) (Contribution 2), who assessed nutrient intake and sensory processing in school-aged children with ASD. Their study focused on nutrients involved in one-carbon metabolism and found associations between specific nutrient intakes and sensory processing domains. Although these findings require confirmation in larger and longitudinal studies, they suggest that dietary assessment in children with ASD should consider not only nutrient adequacy, but also how sensory processing shapes food acceptance, avoidance, and the overall eating experience.

The scope of this Special Issue also extends beyond ASD, incorporating populations with genetic and neuromuscular conditions that present specific feeding-related challenges. De Almeida et al. (2025) (Contribution 3) evaluated food neophobia among Brazilian children with Down syndrome, a genetic condition frequently associated with developmental, sensory, and feeding-related difficulties. Their findings revealed a high prevalence of food neophobia and suggested that the school environment may help encourage acceptance of new foods, particularly fruits. Similarly, Winnicka et al. (2025) (Contribution 4) examined eating and mastication difficulties in children with spinal muscular atrophy (SMA), showing that reduced tongue strength was associated with poorer mastication and swallowing performance. These studies remind us that dietary patterns are shaped not only by preferences, sensory responses, or family practices, but also by oral–motor function, swallowing safety, fatigue, and disease-specific functional limitations.

The review articles included in this Special Issue complement the original studies by integrating current evidence and identifying key gaps for future research. Tomaszek et al. (2025) (Contribution 5) reviewed the connections between eating issues, gastrointestinal symptoms, microbiome alterations, and ASD. Their narrative review highlights a potential cycle in which restricted diets and gastrointestinal discomfort may contribute to microbiota imbalance, which could further influence behavioral symptoms and feeding difficulties. Although evidence in this field remains limited and heterogeneous, this contribution encourages researchers and clinicians to study diet, gastrointestinal health, and microbiome composition as interconnected dimensions.

From a more intervention-oriented perspective, Torres-Collado et al. (2025) (Contribution 6) conducted a scoping review of strategies aimed at promoting fruit and vegetable consumption in children with NDDs. The review identified heterogeneous approaches, including repeated exposure, play-based interventions, behavioral modification, mobile health, dietary approaches, and interdisciplinary mealtime interventions. Overall, parent-led strategies supported by multidisciplinary teams, positive reinforcement, play, and food modification techniques appeared to be the main approaches used to improve fruit and vegetable intake. This review moves the field from description toward action, while also highlighting the need for more rigorous intervention studies with larger samples, longer follow-up periods, standardized outcome measures, and better reporting of acceptability and sustainability.

From a clinical perspective, the articles gathered in this Special Issue reinforce the importance of multidisciplinary teams involving dietitians, pediatricians, occupational therapists, speech and language therapists, psychologists, educators, and families. Nutritional interventions should be developmentally appropriate, culturally sensitive, and adapted to each child’s sensory profile, functional abilities, gastrointestinal symptoms, and family context. From a research perspective, future studies should prioritize longitudinal designs, validated dietary and behavioral assessment tools, objective measures when feasible, and intervention trials that evaluate dietary intake, acceptability, quality of life, caregiver burden, and long-term sustainability.

In conclusion, dietary patterns and feeding challenges in children with NDDs and related conditions cannot be fully understood without considering sensory, behavioral, gastrointestinal, oral–motor, and environmental dimensions. Continued research in this area is essential to design targeted interventions that improve nutritional health, daily functioning, and quality of life for children and their families.
